# Transcriptomic and Metabolomic Networks in the Grape Berry Illustrate That it Takes More Than Flavonoids to Fight Against Ultraviolet Radiation

**DOI:** 10.3389/fpls.2016.01337

**Published:** 2016-08-30

**Authors:** José Tomás Matus

**Affiliations:** Centre for Research in Agricultural Genomics (CRAG), CSIC-IRTA-UAB-UB, BarcelonaSpain

**Keywords:** light, HY5, MYB, flavonols, terpenes, anthocyanins, ripening, stilbene

## Abstract

Plants are constantly challenged by environmental fluctuations. In response, they have developed a wide range of morphological and biochemical adaptations committed to ameliorate the effects of abiotic stress. When exposed to higher solar radiation levels, plants activate the synthesis of a large set of enzymes and secondary metabolites as part of a complex sunscreen and antioxidant defense mechanism. Grapevine (*Vitis vinifera* L.) has become a widely used system for studying adaptive responses to this type of stress since changes in berry composition, positively influenced by increased ultraviolet (UV) radiation levels, improve the quality of wines subsequently produced. Despite the fact that most of the attention has been directed toward the synthesis of flavonoids, recent transcriptomic and metabolomic studies have shown that stilbenoids and isoprenoids (e.g., terpenes and carotenoids) are also an important part of the grape UV-response machinery. This minireview focuses on the latest findings referring to the metabolic responses of grapes to UV radiation and proposes a model for its transcriptional control. Depending on the berry developmental stage and the type of radiation (i.e., irradiance level, exposure length), increased UV levels activate different metabolic pathways through the activity of master regulators belonging to the basic Leucine Zipper Domain (bZIP) and R2R3-MYB transcription factor families. This transcriptional control is influenced by the interaction of other environmental factors such as light, temperature or soil water availability. In grapevine, phenylpropanoids are part of, but are not the whole story, in the fight against radiation damage.

## Introduction

The energy coming from the sun is, at the right dosage, essential for life on Earth. A small fraction of the electromagnetic radiation emitted by the sun is in the ultraviolet (UV) range (100–400 nm). On its way through the atmosphere, UV radiation composition is modified and only a small proportion of its spectrum is transmitted to the Earth’s surface (Frohnmeyer and Staiger, 2003). UV-C (200–280 nm) is completely absorbed by atmospheric oxygen and ozone. The stratospheric ozone layer absorbs only part of UV-B (280–315 nm) and even less of UV-A (315–400 nm; Madronich et al., 1998). Environmental levels of UV radiation may change depending on altitude, latitude, season, daytime, and cloud coverage (McKenzie et al., 2003). However, the progressive reduction of the ozone layer within the last 50 years has generated an increase in the levels of UV radiation (Mackerness, 2000), forcing changes in the life scenario of plant ecosystems.

Due to its high-energy content, UV radiation is capable of inducing mutations when absorbed by DNA. It also inhibits electron transport and collapses membrane permeability, among many other detrimental effects on cellular function (Jenkins, 2009). Plants, as sessile organisms, have evolved and generated diverse adaptive (photomorphogenic) responses to solar UV, leading to morphological and biochemical changes, some of which are common between species (Jiang et al., 2012). For instance, physiological responses to UV-B radiation include altered growth and cell expansion, promotion of branching, shorter petioles and thicker leaf blades (reviewed by Robson et al., 2015). Regarding UV-C, its damage is not physiologically relevant for plants growing in the sun (Stapleton, 1992), but several studies have demonstrated the ability of plants to activate specific responses toward this short-wavelength radiation. Indeed, post-harvest fruits are exposed to short UV-C treatments in order to increase the amount of health-promoting metabolites (Crupi et al., 2013).

UV responses involve large changes in gene expression. These depend on the nature of the radiation, the degree of adaptation/acclimation of the organism, and the interaction with other environmental factors (Jordan, 2002). In fact, one of the main challenges of studying the effects of UV is the difficulty of separating different types of radiation (e.g., UV-A from UV-B), or their effects from those of visible light and temperature. Among the main responses to UV radiation, plants trigger the synthesis of a series of antioxidant and defense-related phytochemicals, many of which are secondary metabolites. Secondary plant compounds promote a plethora of ecological and evolutionary benefits. Among these, some are responsible for the coloring of plant tissues, serving as visual attraction for pollinators and seed dispersers. Secondary metabolites accumulate in plant organs exposed to UV-A, UV-B and UV-C, but the final composition depends on the plant species and may comprise a mixture of several compounds (Jenkins, 2009).

Secondary metabolites give plants a versatile range of UV and excessive light-protective mechanisms, such as attenuation of radiation by filtering and antioxidant activity due to the scavenging of free radicals (Tattini et al., 2015). In addition, the accumulation of these compounds positively affects the overall response of plants to biotic stresses. For instance, increasing UV-B in *Arabidopsis* generates a phenolic-based resistance toward the fungus *Botrytis cinerea* through the activation of the phenylpropanoid biosynthetic pathway, in a process dependent on UV-B perception and signaling (Demkura and Ballaré, 2012).

**FIGURE 1:**
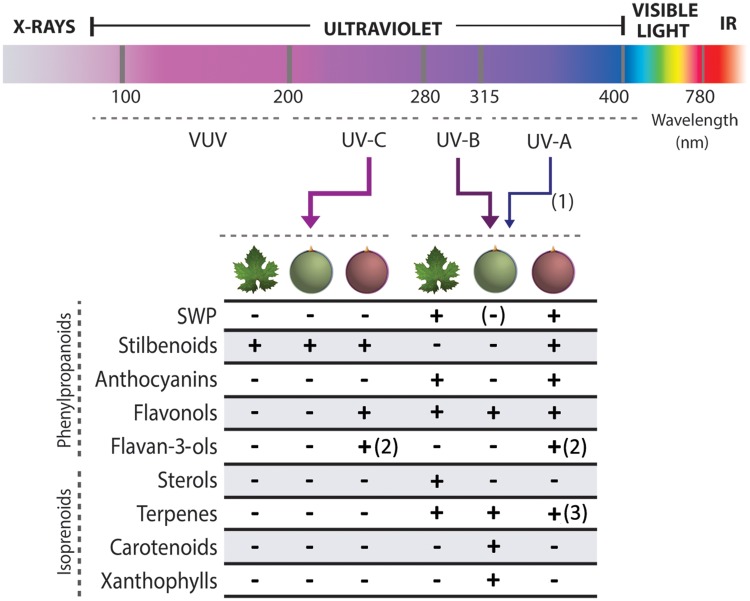
**Secondary metabolites induced in grapevine leaves and fruits in response to ultraviolet radiation.** Red and green berry colors represent black and white-skinned cultivars, respectively. (1): The thickness of the arrows represents the importance of each type of radiation in the corresponding studies. Some of these were not able to differentiate UV-A from UV-B effects as broadband lamps were used. (2): Zhang et al. (2013) showed that flavan-3-ols (proanthocyanidin monomers) were induced by UV-A, B and C but that these increases were not translated into the final ripening stage, probably due to polymerization into condensed tannins or by reversion into other pathways. (3): Accumulation predicted from high expression of *TERPENE SYNTHASES* (Carbonell-Bejerano et al., 2014). (-): Accumulation of small weight phenolic compounds (SWP) shows different tendencies depending on the cultivar and organ (Kolb et al., 2001, 2003). VUV: Vacuum UV. IR: Infrared. ‘-’: Means not tested (in targeted analyses) or not detected (in untargeted metabolomic studies).

Grapevine (*Vitis vinifera* L.) is one of the most extensively cultivated fruit crops in the world. It is considered to be a well-adapted species to UV, and a relevant model for studying adaptive radiation responses. It constitutes an important source of secondary metabolites, mainly derived from the phenylpropanoid and isoprenoid/cholesterol biosynthetic pathways, and their synthesis is enhanced by all types of UV radiation (**Figure 1**). Several studies have furthered our understanding of the effects of UV radiation on the physiology of the vine and in the metabolic response in vegetative and reproductive organs. Furthermore, the sequenced genome of *V. vinifera* has allowed the identification of a few *Arabidopsis* ortholog genes that play a part in the UV signaling pathway, promoting the activation of secondary metabolism (Loyola et al., 2016). The contributions of large-scale analyses, mainly transcriptomic and metabolomic, in understanding the regulatory networks that control UV responses have been the subject of several studies (Pontin et al., 2010; Carbonell-Bejerano et al., 2014; Suzuki et al., 2015; Joubert et al., 2016). The focus of the following minireview is on the grape berry transcriptional mechanisms that relate phenylpropanoid and isoprenoid accumulation to UV-signaling. The most recent genome-wide studies and transcription factor gene characterizations are also discussed.

## A Metabolic Grid of Diverse Compounds Induced by Different Types of Radiation

### Phenylpropanoids

Phenylpropanoids are synthesized as part of the phenylpropanoid pathway. Their numerous functional features have permitted the adaptation and evolution of plants by participating in several essential processes for development and adaptation to the environment (reviewed by Shirley, 1996). In grapes, these compounds are classified into four main groups: small-weight phenolic acids (SWP), lignins, stilbenoids and flavonoids, all of which have been related (directly or indirectly) to UV radiation responses. The first group is characterized by a single ring of 6 carbon atoms (C6) such as benzene (C6-C1) and hydroxycinnamic acids (C6-C3). Stilbenes (C6-C2-C6) are anti-microbial compounds largely studied for their anti-inflammatory and anti-carcinogenic properties in mammals (e.g., resveratrol, Neves et al., 2012). Flavonoids (C6-C3-C6) constitute one of the most abundant groups in the grape berry and consist of a mixture of different glycosylated and acylated derivatives of anthocyanins, proanthocyanidins (PAs) and flavonols.

This phenylpropanoid pathway is one of the most studied in the plant kingdom, both at the enzymatic and regulatory levels, and its modulation by UV radiation has been extensively investigated in grapevine organs. The phenylpropanoid pathway demands high energy expenses for plant cells; not only with respect to their synthesis and transcriptional regulation, but also regarding their final destination in sub-cellular compartments. The synthesis of these compounds must therefore respond to specific developmental cues and also to the environment. Solar radiation has a profound effect on phenylpropanoid accumulation due to the synergic effects of the visible light spectra and the UV range of wavelengths. Different microclimatic light conditions, manipulated in the area surrounding the grape bunches by means of leaf displacement or removal, increase the accumulation of flavonols and anthocyanins at ripening (Matus et al., 2009). However, this response seems to be cultivar-specific (Pastore et al., 2013). Several studies have differentiated the effects of UV radiation and light on the accumulation of phenylpropanoids (**Figure 1**). These will now be described in more detail.

UV-B radiation has a particularly strong effect on the synthesis of flavonols in the grape berry skin. Loyola et al. (2016) performed UV-B inductive experiments in grapevine plantlets and fruits from 9 year-old cv. Cabernet Sauvignon potted vines, by using a combination of broadband UV-B lamps and polyester filters that specifically absorbed the UV-B spectra (used as -UV controls). This work showed that flavonols were highly induced by UV-B, in correlation with a higher expression of their structural and regulatory genes. Using a complementary approach, Carbonell-Bejerano et al. (2014) performed UV radiation blocking and transmitting experiments in commercial vineyards of cv. Tempranillo, finding that solar UV radiation was necessary to enhance phenylpropanoid accumulation at later fruit ripening stages. The authors performed a microarray analysis, and identified more than 120 genes within the berry skin transcriptome that were significantly altered by UV (UV-A and UV-B radiation effects were not distinguished in this study). Some of the most differentially expressed transcripts belonged to light-signaling processes and to the flavonol-related branch. These transcriptomic results correlated with the flavonol profiles obtained from the same samples. In addition, and even though total anthocyanin amounts didn’t changed significantly, the amount of some derivatives, such as petunidin-3-*O*-(6′-acetyl) and delphinidin-3-*O*-(6′-*p*-coumaroyl) glucosides, were higher in non-filtered UV-B conditions. At the end of ripening, the authors also found a few SWPs were induced by UV-B, such as *p*-coumaric acid.

UV-induced changes in anthocyanin abundance and composition in the grape berry were also observed by Martínez-Lüscher et al. (2014). The authors observed an interaction of UV-B and water deficit, an association possibly related to the hormone abscisic acid (ABA), as proposed by Berli et al. (2011). In addition to the anthocyanin and flavonol pathways, the stilbene branch also seems to be transcriptionally modulated in response to UV-B (**Figure 1**), as shown by Carbonell-Bejerano et al. (2014). However, UV-C radiation preferentially induces this branch in comparison to the flavonoid pathway (Suzuki et al., 2015). Stilbenoids are antimicrobial compounds that rapidly accumulate in grapevine defense responses toward fungal diseases such as powdery or downy mildew and gray mold (reviewed by Vannozzi et al., 2012). In addition to their activity as phytoalexins against these pathogens, stilbenoids accumulate in response to wounding (Pezet et al., 2003), UV-C exposure (Adrian et al., 2000) and the hormone methyl jasmonate (Vezzulli et al., 2007; Almagro et al., 2014). Their synthesis depends on the activity of STILBENE SYNTHASES (STS), the archetypal enzyme of CHALCONE SYNTHASES (CHS) as they compete each other for the same phenylpropanoid intermediate, *p*-coumaroyl-CoA. Vannozzi et al. (2012) and Parage et al. (2012) showed that the STS family in grapevine has expanded extensively by tandem duplications, with more than 30 members found to date. When vegetative grapevine organs were challenged with UV-C treatments, many of these *STS*s were rapidly activated, in an opposite fashion to *CHS* expression (Vannozzi et al., 2012).

Regarding grapevine fruits, Suzuki et al. (2015) analyzed the transcriptome and metabolome changes in berry skins exposed to UV-C radiation, finding 238 up-regulated genes classified in gene ontology terms such as pectinesterase, chlorophyllase, and resveratrol synthetic activities. The authors indeed showed a clear induction of many *STS* genes by UV-C, in correlation with an increase in *trans*-resveratrol, ε-viniferin and piceid concentrations. Interestingly, anthocyanins, PAs or flavonols were little affected when comparing dark and UV-C treated fruits.

### Sterols and Isoprenoids

Compounds derived from the cholesterol and isoprenoid biosynthetic pathways have been recently shown to form part of the radiation responses in grapevine leaves (Gil et al., 2012) and berries (Young et al., 2016). These compounds include different types of sterols and phosphate derivatives of isopentenyl, geranyl, farnesyl, geranylgeranyl, and presqualene. They act as accessory pigments for light harvesting (e.g., carotenoids), radical scavengers (e.g., tocopherols), membrane components (e.g., sterols), volatile aroma compounds (e.g., mono- and sesquiterpenes) as well as phytohormones (e.g., ABA).

The biosynthesis of isoprenoids in grapes occurs through two physically separated but tightly connected pathways: the methylerythritol phosphate (MEP) and mevalonate (MVA) pathways (Luan and Wüst, 2002). Both pathways are responsible for the production of volatile terpenes. The plastid-localized MEP is the predominant pathway for synthesis of monoterpenes and diterpenes, while the cytosolic MEV is mainly responsible for sesquiterpene production (Bohlmann and Keeling, 2008). Grapevine possesses a large family of terpene synthases (TPS), with more than 100 members functionally validated to date (Martin et al., 2010).

Gil et al. (2012) found that low UV-B exposures in vegetative grapevine tissues increased the levels of the membrane-related sitosterol, stigmasterol and lupeol, therefore proposing that UV-B induced the synthesis of terpenes involved in membrane stability. In addition, they found that high UV exposure induced defense mechanisms related to the antioxidant capacity (i.e., accumulation of the diterpenes tocopherol and phytol) and generated a rapid and transient accumulation of the sesquiterpenic stress-related hormone ABA. TPS activity, as observed by the accumulation of monoterpenes and sesquiterpenes, was induced by both low and high radiations. By using microarray tools it become apparent that a much broader transcriptional reprogramming of the terpene biosynthetic pathway occurred in berries in response to UV-B (in addition to the activation of the stilbene and flavonol pathways as described earlier in this minireview; Carbonell-Bejerano et al., 2014). Several genes coding for *TPS* were induced by UV at late ripening stages in comparison to UV filtered fruits. Unfortunately, metabolic quantification of terpenes was not performed to correlate these results.

Some recent metabolic studies have gone deeper into the relation of light and UV exposure with the volatile composition of grapes and wines. Song et al. (2015) showed that higher bunch exposures influenced the accumulation of terpene alcohols, C13-norisprenoids and other volatiles in cv. Pinot noir wines. The content of the monoterpene linalool and the expression of its biosynthetic genes were reduced in shaded or UV-filtered berries of cv. Riesling (Sasaki et al., 2016). The authors also showed that light reflection was sufficient for an increase in linalool concentration. Recently, Joubert et al. (2016) used a metabolomic approach to show that in addition to volatile aroma compounds, UV-B could also increase the accumulation of carotenoids and xanthophylls in white skinned berries of cv. Sauvignon blanc (**Figure 1**). The authors hypothesized that these molecules could act as acclimation plasticity factors, by mitigating photoinhibition and photodamage. Additionally, the increase in aromatic compounds could be directly related to the accumulation of carotenoids as these serve as substrates for the formation of norisoprenoids. Further transcriptomic analyses may be needed to correlate this metabolic reprogramming with the expression of genes from the MEP and MVA pathways. In addition, these future studies could identify transcriptional regulators of their synthesis. Recently, Wen et al. (2015) proposed several transcription factors as candidate regulators of terpene accumulation. The authors performed RNA-seq and gene network analyses to correlate gene co-expression with the differential accumulation of terpenes across two viticultural regions with contrasting temperatures. Genes belonging to the Trihelix, MYB, WERK, and ERF families were found associated with these changes.

## Transcriptional Regulation of UV-Induced Metabolic Responses

### R2R3-MYB Transcription Factors as Immediate Regulators of Secondary Metabolism

The activation of phenylpropanoid-related genes is achieved by transcription factors belonging to several protein families. The largest breakthrough has emerged from the study of the combinatorial interaction between R2R3-MYB, beta helix-loop-helix (bHLH) and tryptophan and aspartic acid rich repeat (WDR) proteins (reviewed in grapevine by Hichri et al., 2011).

R2R3-MYB proteins belong to the biggest transcription factor Superfamily described in plants (the MYB Superfamily) and are characterized by two highly conserved DNA binding domains and a variant number of C-terminal motifs (Dubos et al., 2010). Matus et al. (2008) showed that grapevine R2R3-MYB genes related to phenylpropanoid regulation have expanded by duplication. To date, the number of grape genes belonging to this family is 134 (Wong et al., 2016). Several members of the family have been characterized, many of which act as positive regulators of the synthesis of anthocyanins (MYBA1/A2, MYB5B), PAs (MYBPA1/PA2/PAR), and flavonols (MYBF1). More recently, repressors of the accumulation of SWPs and flavonoids (Cavallini et al., 2015) and positive regulators of stilbenoid accumulation (MYB15 and MYB14; Höll et al., 2013) have also been identified.

Several studies have documented that the characterized grape R2R3-MYB genes respond to UV radiation (Pontin et al., 2010; Höll et al., 2013; Zhang et al., 2013; Carbonell-Bejerano et al., 2014; Cavallini et al., 2015; Loyola et al., 2016; Wong et al., 2016). The general tendency is for MYB activators to increase their expression on irradiation, whereas the opposite behavior occurs for phenylpropanoid repressors. R2R3-MYB repressors belonging to Subgroup 4 (also known as MYB-C2) fine-tune the balance of the different branches of the pathway. In particular, VviMYB4A has an important role in the acclimation and adaptation of vegetative tissues to UV-B radiation (Cavallini et al., 2015). Regarding stilbene pathway regulation, Wong et al. (2016) recently identified a close homolog of MYB14 and MYB15, also belonging to Subgroup 2. MYB13 was identified as an additional *STS* regulator by coupling gene co-expression networks and the search of MYB binding sites in the promoter regions of co-expressed genes (Wong et al., 2016). As expected, MYB13/14/15 were induced by UV-C treatments, although in different intensities and at different time points of the response.

The reanalysis of the grape R2R3-MYB family showed that many members participate in secondary metabolic processes and that this regulation goes beyond the phenylpropanoid pathway. Representation analysis of the MapMan BIN terms for their TOP100 co-expressed genes showed that several MYB genes were highly associated with terpenes (Wong et al., 2016). Thus, it is expected that some of these may directly regulate *TPS* or other genes of the isoprenoid pathway, as occurs with flavonoids or stilbenoids. One certainly interesting candidate is MYB24, one of the most UV-induced genes in the study of Carbonell-Bejerano et al. (2014). This gene was recently associated with terpene synthesis in berries in response to drought (Savoi et al., 2016) and is highly co-expressed with flower and fruit specific *TPS* (Wong et al., 2016).

### UV-B Signaling Factors from the bZIP Family Orchestrate UV Secondary Metabolic Responses

The proteins involved in the UV signal transduction pathway and the enzymes related to flavonoid biosynthesis may have evolved together in the water-to-land transition, due to higher exposure to UV-B radiation. The absence of flavonoids in algal species and the synthesis of flavonols in response to UV-B in the moss *Physcomitrella patens* supports this hypothesis (Wolf et al., 2010).

In *Arabidopsis*, two general signaling pathways exist for responding to UV-B (Jenkins, 2009): (1) a non-specific pathway, triggered by high levels of radiation (causing direct DNA damage and release of reactive oxygen species) and (2) a photomorphogenic signaling pathway that responds to low levels of UV-B radiation. In the latter, UV-B is perceived by the UV-B receptor UV RESISTANCE LOCUS 8 (UVR8; Rizzini et al., 2011) which in turn activates a signaling cascade mediated by ELONGATED HYPOCOTYL 5 (HY5; Brown et al., 2005). AtHY5 is a master regulator of several plant processes including hypocotyl growth in the dark (Ulm et al., 2004), nitrate uptake in roots (Chen et al., 2016) and regulation of flavonol and anthocyanin synthesis in response to light and UV radiation (Stracke et al., 2010; Shin et al., 2013). In tomato, a central role of LeHY5 has being assigned to the regulation of carotenoid genes in coordination with PIF proteins (reviewed by Llorente et al., 2016).

Loyola et al. (2016) reported the characterization of the grape UVR8 (VviUVR1) and HY5 (VviHY5 and VviHYH) orthologs, demonstrating their role in flavonol regulation in leaves and berries upon UV-B radiation. A comprehensive analysis of co-expression and genome-wide VviHY5 binding sites was conducted and tested with a transient expression assay in grape, demonstrating its capacity to induce the expression of flavonol-structural and regulatory genes. As seen from this work, the berry UV-B response machinery favors flavonol accumulation by activating *VviHY5* and *VviHYH* at pre and post-veraison berry developmental stages, respectively, in high and low UV-B exposures. A similar UV-responsive behavior was obtained for *VviHY5* in white-skinned berries of cv. Sauvignon blanc (Liu et al., 2014). The expression and role of HY5 resembles that of VvibZIPC22, a flavonoid-regulator recently characterized by Malacarne et al. (2016), which is induced by UV treatments in leaves of cv. Chardonnay. As suggested by the authors, the regulation of the phenylpropanoid pathway by bZIP factors may span different levels: (1) by forming dimers with other bZIP proteins or by interacting with non-bZIP proteins required for the regulation of structural target genes and (2) by directly targeting the regulators (i.e., by activating *R2R3-MYB* and *bZIP* genes). VvibZIPC22 does not appear in the VviHY5 co-expression catalog (Loyola et al., 2016). However, this does not exclude the possibility of indirect regulation or protein interaction. For instance, AtHY5 lacks an activation domain and it does not activate expression by itself but through interacting with other proteins (reviewed by Stracke et al., 2010).

**FIGURE 2:**
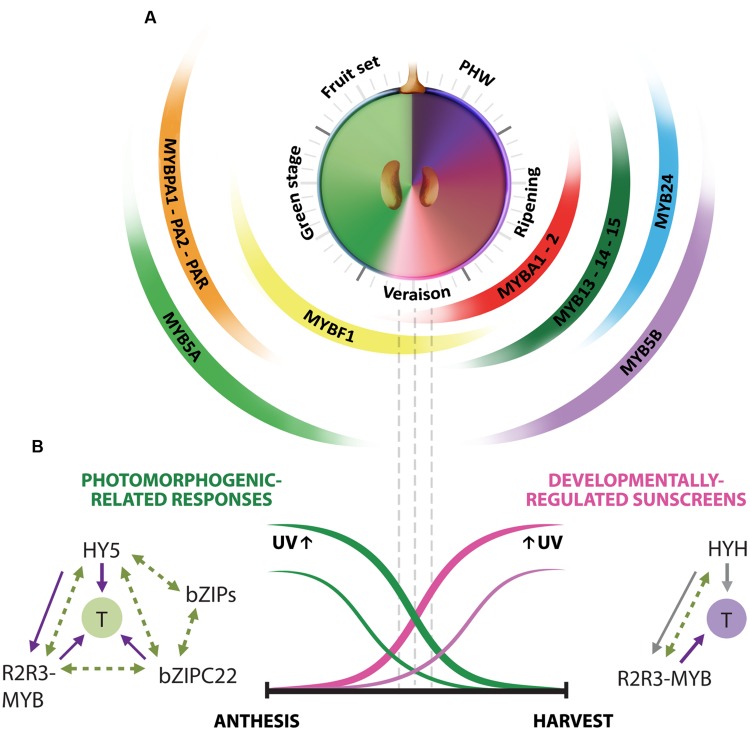
**UV-responsive regulatory networks controlled by R2R3-MYB and bZIP transcription factors are hierarchized by developmental stage in the black-skinned grape berry. (A)** Multi-layered transcriptional regulation of secondary metabolism exerted by R2R3-MYB factors in the grape berry. Berry development is described in resemblance to a clock, with the fruit set–*to*–post harvest-withering (PHW) stages shown in anticlockwise direction. Veraison corresponds to the onset of ripening. The secondary metabolic pathways controlled by these regulators are: PAs or proanthocyanidins (MYBPA1/PA2/PAR), early steps of the flavonoid branch (MYB5A), anthocyanins (MYBA1/A2 and to a lesser extent, MYB5B), flavonols (MYBF1), stilbenoids (MYB13/14/15) and terpenes (putatively assigned to MYB24). MYBC2-repressors also respond to UV but they were not included, as they haven’t been tested in grape berries exposed to UV. **(B)** Orchestration of UV responses by bZIP transcription factors. The HY5-photomorphogenic and bZIPC22-related networks are induced by UV and act mainly at pre-veraison stages. The response of HYH to UV occurs predominantly at ripening, concomitant with the developmentally regulated accumulation of natural sunscreens. bZIP factors regulate flavonoid synthesis by activating both R2R3-MYB regulators and their structural targets (T). The complementary expression patterns of *HY5* and *HYH* in development and their induction by both low and high UV intensities is hypothesized to be in part responsible for the successful adaptation of this species to radiation. Solid lines represent directly tested (purple) or hypothesized (gray) gene expression regulation. Dashed lines represent putative protein–protein interactions.

From all the studies gathered in this minireview, it is possible to suggest a model describing the transcriptional regulation of secondary metabolism in response to UV radiation (**Figure 2**). In grapes, UV can simultaneously reprogram at least three secondary metabolic branches: flavonoids, stilbenes and terpenes, by activating the direct R2R3-MYB regulators of these pathways. The UV-B adaptive mechanisms that grapevines possess are highly efficient in part due to the activation of bZIP regulators in response to high UV-B exposures, in addition to their conserved photomorphogenic response to low radiation exposures. These bZIP factors orchestrate MYB-mediated responses by activating their expression, and also by directly regulating the structural genes of the flavonoid pathway. Whether these factors also regulate terpenes and stilbenoids, still needs to be further tested. Finally, as all these secondary metabolic routes are highly demanding for energy and carbon compounds, and since all of them are induced in response to UV, it is necessary to determine if these pathways somehow compete between each other. A certainly interesting approximation would be to study source-to-sink relationships and carbon fluxes between these pathways.

## Author Contribution

JM searched and discussed the literature and wrote the article.

## Conflict of Interest Statement

The author declares that the research was conducted in the absence of any commercial or financial relationships that could be construed as a potential conflict of interest.
